# Supplementary material for: Monocyte-lymphocyte cross-communication via soluble CD163 directly links innate immune system activation and adaptive immune system suppression following ischemic stroke
Source: Sci Rep. 2017 Oct 11;7:12940. doi: 10.1038/s41598-017-13291-6 (PMC5636885; doi:10.1038/s41598-017-13291-6)
Supplement: Supplementary file 1 — Supplementary Materials [file 41598_2017_13291_MOESM1_ESM.pdf]

SUPPLEMENTARY MATERIALS

Monocyte-lymphocyte cross-communication via soluble CD163 directly links innate immune system activation and adaptive immune system suppression following ischemic stroke.

Grant C. O’Connell, PhD; Connie S. Tennant, RN; Noelle Lucke-Wold, RN; Yasser Kabbani, MD; Abdul R. Tarabishy, MD; Paul D. Chantler, PhD; Taura L. Barr, RN, PhD

Supplementary Tables: 3
Supplementary Figures: 3

Supplementary Table 1. **Demographic characteristics of healthy leukocyte donors.**

	n=12
**Age *(mean ± SD)***	25.5 ± 3.9
**Female *n (%)***	6 (50.0)
**History of cardiovascular disease *n (%)***	0 (0.00)
**History of autoimmune disease *n (%)***	0 (0.00)
**History of cancer *n (%)***	0 (0.00)
**Prior or current pregnancy *n (%)***	0 (0.00)
**History of head injury *n (%)***	0 (0.00)
**History of neurological disorder *n (%)***	0 (0.00)
**Current smoker *n (%)***	0 (0.00)

Supplementary Table 2. **Clinical and demographic characteristics of subject sub-populations used for independant replicate in vitro experiments.**

	Group:			p-value:		
	Asymptomatic (A; n=10)	Stroke Mimic (SM; n=10)	Ischemic Stroke (IS; n=10)	Main Test	A vs. IS	SM vs. IS
**[a]Age *(mean ± SD)***	59.7 ± 10.5	59.6 ± 15.9	64.8 ± 8.8	0.589	-	-
**[b]Female *n (%)***	7 (70.0)	4 (40.0)	4 (40.0)	0.467	-	-
**[a]NIHSS *(mean ± SD)***	0.0 ± 0.0	6.9 ± 4.9	5.9 ± 3.7	<0.001*	<0.001*	1.000
**[b]Family history of stroke *n (%)***	6 (60.0)	3 (30.0)	6 (60.0)	0.467	-	-
**[b]Hypertension *n (%)***	6 (60.0)	9 (90.0)	8 (80.0)	0.430	-	-
**[b]Dyslipidemia *n (%)***	3 (30.0)	6 (60.0)	5 (50.0)	0.534	-	-
**[b]Diabetes *n (%)***	2 (20.0)	6 (60.0)	5 (50.0)	0.266	-	-
**[b]Previous stroke *n (%)***	0 (00.0)	4 (40.0)	0 (00.0)	0.023*	1.000	0.173
**[b]Atrial fibrillation *n (%)***	0 (00.0)	1 (10.0)	1 (10.0)	1.000	-	-
**[b]Myocardial infarction *n (%)***	0 (00.0)	5 (50.0)	4 (40.0)	0.038*	0.173	1.000
**[b]Hypertension medication *n (%)***	5 (50.0)	8 (80.0)	6 (60.0)	0.510	-	-
**[b]Diabetes medication *n (%)***	1 (10.0)	5 (50.0)	5 (50.0)	0.124	-	-
**[b]Cholesterol medication *n (%)***	4 (40.0)	5 (50.0)	5 (50.0)	1.000	-	-
**[b]Anticoagulant or antiplatelet *n (%)***	0 (00.0)	7 (70.0)	5 (50.0)	0.004*	0.065	1.000
**[b]Current smoker *n (%)***	0 (00.0)	1 (10.0)	1 (10.0)	1.000	-	-

[a]Means compared via one-way ANOVA with subsequent planned group-wise comparisons using Bonferroni-corrected two-sample two-tailed t-test; [b]Proportions compared via 2x3 Fisher's exact test with subsequent planned group-wise comparisons using Bonferroni-corrected 2x2 Fisher's exact test; NIHSS, National Institutes of Health stroke scale; SD, standard deviation; *p<0.05

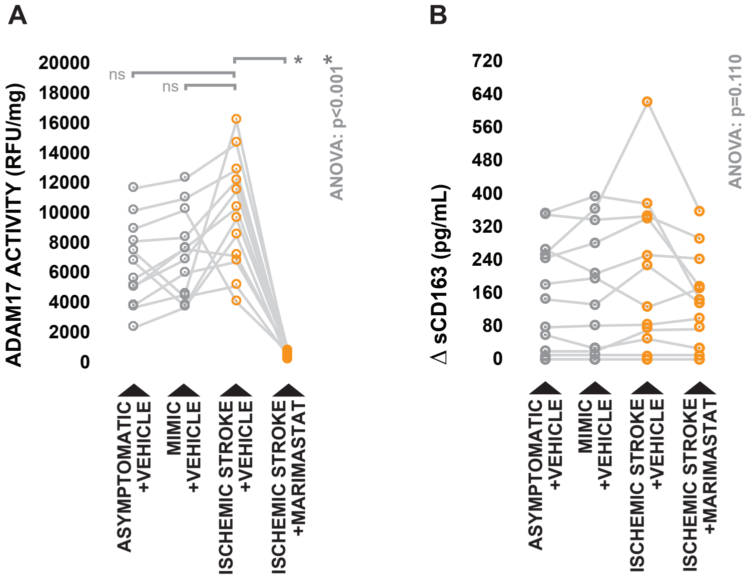

Supplementary Figure 1. Effects of ischemic stroke serum on neutrophil ADAM17-dependant sCD163 production in an independent replicate experiment. (A) ADAM17 activity in healthy-donor neutrophil lysates collected following three hours incubation with 10% serum obtained from AIS patients and control subjects, in either the presence and absence of ADAM17 inhibitor. (B) Neutrophil-derived sCD163 levels in cell culture supernatants collected following treatment, presented as the difference in sCD163 levels observed between cell culture supernatants and serum-supplemented media incubated in the absence of cells. Means were compared via repeated measures one-way ANOVA; the Greenhouse-Geisser correction was applied to account for non-sphericity. In the case of a significant test, subsequent planned group-wise comparisons were performed using Bonferroni-corrected paired two-tailed t-test; planned comparisons are indicated by brackets.

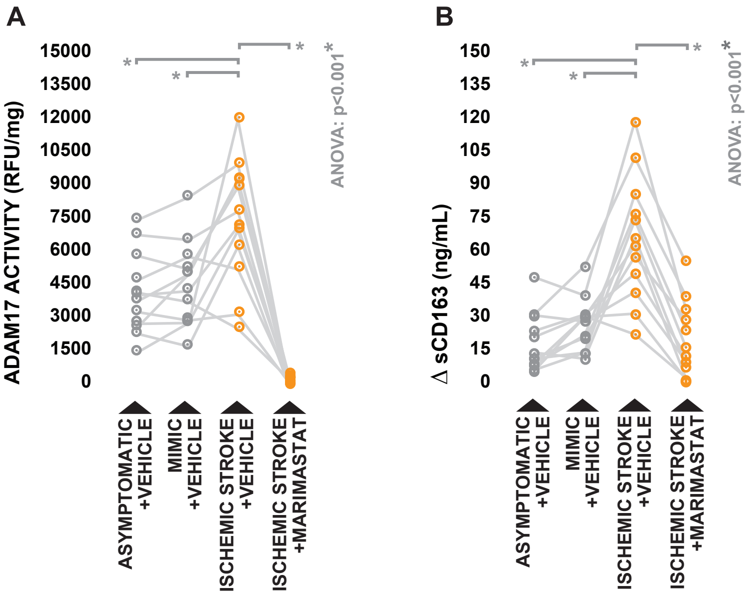

Supplementary Figure 2. Effects of ischemic stroke serum on monocyte ADAM17-dependant sCD163 production in an independent replicate experiment. (A) ADAM17 activity in healthy-donor monocyte lysates collected following three hours incubation with 10% serum obtained from AIS patients and control subjects, in either the presence and absence of ADAM17 inhibitor. (B) Monocyte-derived sCD163 levels in cell culture supernatants collected following treatment, presented as the difference in sCD163 levels observed between cell culture supernatants and serum-supplemented media incubated in the absence of cells. Means were compared via repeated measures one-way ANOVA; the Greenhouse-Geisser correction was applied to account for non-sphericity. In the case of a significant test, subsequent planned group-wise comparisons were performed using Bonferroni-corrected paired two-tailed t-test; planned comparisons are indicated by brackets.

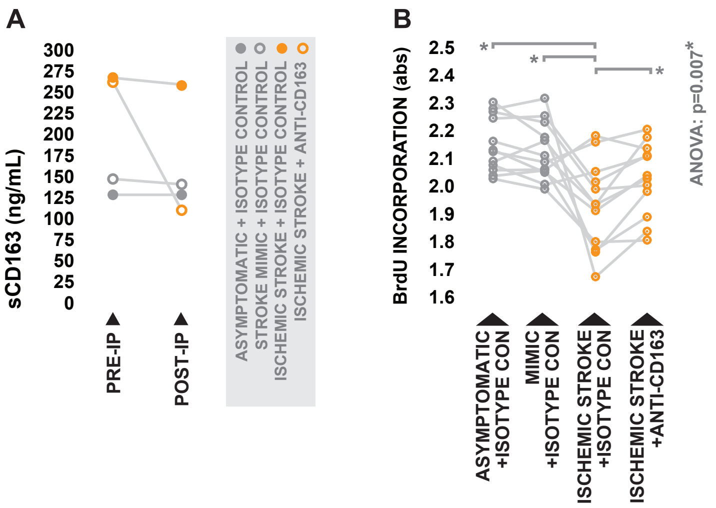

Supplementary Figure 3. Influence of post-stroke peripheral blood sCD163 levels on the capacity to support lymphocyte proliferation in an independent replicate experiment. (A) Pre and post-immunoprecipitation concentrations of sCD163 in pooled serum samples obtained from ischemic stroke patients and controls, immunoprecipitated using either anti-CD163 polyclonal antibody or isotype control. (B) BrdU incorporation in healthy donor-derived lymphocytes over the final 24 of 72 hours PHA-stimulated proliferation in the presence of 20% post-immunoprecipitation pooled serum. Means were compared via repeated measures one-way ANOVA; the Greenhouse-Geisser correction was applied to account for non-sphericity. In the case of a significant test, subsequent planned group-wise comparisons were performed using Bonferroni-corrected paired two-tailed t-test; planned comparisons are indicated by brackets.

Supplementary Table 3. Primers and thermocycling conditions used for qRT-PCR.

Gene	Transcripts[a]	Primers (5' to 3')[b]		Product (bp)
ADAM17	NM_003183.5	**FOR:**	GTGGATGGTAAAAACGAAAGCG	**93**
	XM_017004786.1	**REV:**	GGCTAGAACCCTAGAGTCAGG	
	XM_017004785.1			
	XM_011510376.2			
	XM_011510375.2			
CD163	NM_004244.5	**FOR:**	GCGGGAGAGTGGAAGTGAAAG	**89**
	XM_005253529.3	**REV:**	GTTACAAATCACAGAGACCGCT	
	XM_005253528.3			
	NM_203416.3			
PPIB	NM_000942.4	**FOR:**	AAGTCACCGTCAAGGTGTATTTT	**153**
		**REV:**	TGCTGTTTTTGTAGCCAAATCCT	
B2M	NM_004048.2	**FOR:**	GAGGCTATCCAGCGTACTCCA	**248**
	XM_006725182.2	**REV:**	CGGCAGGCATACTCATCTTTT	
	XM_005254549.2			
ACTB	NM_001101.3	**FOR:**	CATGTACGTTGCTATCCAGGC	**250**
	XM_006715764.1	**REV:**	CTCCTTAATGTCACGCACGAT	

[a]Listed by NCBI accession number; [b]All targets were amplified for 40 cycles of 95ºC (15s) / 60ºC (60s)
